# Bandwidth Cost Minimization via User Association for Enterprise WLANs

**DOI:** 10.3390/s18124104

**Published:** 2018-11-23

**Authors:** Xili Wan, Xinjie Guan, Wentian Zhao, Guangwei Bai, Baek-Young Choi

**Affiliations:** 1 School of Computer Science and Technology, Nanjing Tech University, Nanjing 211816, China; xiliwan@njtech.edu.cn (X.W.); 158016079@njtech.edu.cn (W.Z.); bai@njtech.edu.cn (G.B.); 2 Department of Computer Science Electrical Engineering, University of Missouri-Kansas City, Kansas City, MO 64110, USA; choiby@umkc.edu

**Keywords:** bandwidth cost, user association, enterprise WLANs, approximation algorithm

## Abstract

Enterprise Wireless LANs (E-WLANs) such as airport WiFi, have become a convenient way for Internet access for mobile users. In an E-WLAN, access points (APs) are usually deployed with high-density around the infrastructure to provide sufficient coverage and for a better service, where a mobile user chooses one AP to associate with among multiple available APs in the vicinity. Many studies have been done on developing user association techniques to increase system performance, with various objectives including network throughput maximization, load balancing etc. Our work is unique in that we focused on bandwidth cost minimization via user association from the perspective of the E-WLAN operators. Specifically, by considering the bandwidth demands from mobile users, we modeled the joint user association and cost minimization problem in the heterogeneous E-WLAN with additional constraints from individual bandwidth demands as an optimization problem. To solve the optimization problem efficiently, we propose an approximation algorithm using relaxation and rounding techniques. We prove that the proposed algorithm has performance bound with a constant ratio to the optimization problem. Furthermore, our simulation results exhibit the superiority of our proposed algorithm over prior schemes.

## 1. Introduction

Due to the convenience of mobility and easy set-up, Wireless Local Area Networks (WLANs), mostly based on WiFi, has become a conventional way for the Internet access to mobile users. WLANs have been widely deployed in various scenarios from home wireless networks to enterprise networks. Moreover, it could be adapted to Internet-of-Things scenarios [1]. The popularity of smart devices further promotes the wide deployment of WLANs, especially in enterprise networks. For example, airport WiFi has become the dominant way for air passengers to access the Internet while waiting for their flights. To provide a better quality of service, access points (APs) are usually deployed with high-density within the enterprise infrastructure, where each mobile user has multiple APs to choose for an association. With the explosive growth of mobile devices, many challenges have been brought to WLANs in enterprise networks. User association is one of the challenging problems, which is to determine one-to-one pair between mobile users and proper APs for achieving high network performance, in various network sites including airports, university campuses and large public places such as stadiums and malls where high-density APs are deployed for a large number of mobile users.

The default association method for a mobile user is defined by the 802.11 protocol which is to associate an AP with the strongest Received Signal Strength Indicator (RSSI) value. The use of RSSI has been widely used for indoor localization [2] as well. However, this default mechanism may lead to imbalance of loads on APs and decreased network throughput [3]. To enhance the performance, much work has been conducted on user association with the objectives, such as the maximization of network load balancing or total network throughput [4,5,6]. There are also studies that aim to maximize the utilization of overall bandwidth, from the view of the system level at the time of user association [7,8,9].

However, these studies do not consider each user’s bandwidth demand. In fact, each mobile user tends to have their own bandwidth requirements for various applications. For example, in an airport, a passenger who wants to stream a high-definition movie must have a larger bandwidth demand than that of browsing online news. Without considering the users’ bandwidth demands, the allocated bandwidth could be insufficient or wasteful for associated users. Moreover, in some enterprise WLAN (E-WLAN) such as an airport WiFi, users may be charged based on their bandwidth demands. This makes mobile users choose the proper bandwidth demand according to their demands.

Most importantly, for the E-WLAN providers, one major goal is to minimize the cost of operating the WLANs. Among the overall cost, bandwidth cost is one of the major costs of maintaining the E-WLANs and it is proportional to the amount of used bandwidth. From the perspective of the E-WLANs providers, it is essential to minimize the total bandwidth cost.

Motivated by this, we identify the joint user association and cost minimization problem for E-WLANs, while satisfying the bandwidth demands specified by mobile users. We refer this problem as the Bandwidth Cost Minimization for Association (BCMA) problem in this paper. As an initial work in this direction, we focus on bandwidth cost minimization while considering the users’ bandwidth demand constraints. We leave the considerations of load balancing and fairness as our future work.

We then formulate this BCMA problem, as an optimization problem. However, the BCMA problem is hard to solve directly as it involves binary constraints. Typical heuristic algorithms cannot provide a guaranteed performance. That is, it is not clear how good or bad the solution from the heuristic algorithm is compared to the optimal solution. To achieve a solution with a guaranteed performance bound, we seek to design an approximation algorithm for the BCMA problem by adapting the relaxation and rounding techniques, to mitigate the involved binary constraints. Unlike conventional rounding techniques, we uniquely extend bipartite rounding techniques to graph modeling, which helps us design the specific algorithm with performance guarantee.

The contributions of this paper are summarized as follows.

(1) We formulate the user association and bandwidth cost minimization that considers users’ bandwidth demands, as an optimization problem.
(2) To solve the problem, we develop an approximation algorithm by extending the integer relaxing and the bipartite rounding technique. Specifically, to bound the integrality gap induced by relaxation, we design a specific function and introduce it into the objective function. Moreover, we adapt the bipartite rounding by constructing a special bipartite graph with auxiliary nodes to represent the potential association. Then the min-cost flow algorithm is applied to determine the final association which considers the users’ demands.
(3) By exploiting the integrality gap and the properties of min-cost flow, we theoretically prove that the proposed algorithm could always achieve an approximation ratio of 2. Moreover, we evaluate our algorithm with extensive simulations in terms of throughput, the total min-cost and the user acceptance ratio under uniform and non-uniform distribution. Furthermore, simulation results have exhibit the performance superiority of the proposed algorithm over existing methods with respect to cost, throughput and acceptance ratio.

The remainder of this paper is structured as follows. Section 2 introduces the related work on the joint user association and bandwidth cost minimization problem. Section 3 defines the system model. Section 4 presents our approximation algorithm for the BCMA problem and proves its approximation ratio. Simulation results are given in Section 5. Finally, we conclude the paper in Section 6.

## 2. Related Work

WiFi has been broadly studied from various aspects, e.g., fast connection [1], indoor localization [2,10]. However, the default association method for a mobile user in 802.11 protocol is not an efficient approach for user-to-AP association as proved in [11], therefore various studies have been performed to properly associate users to APs for different purposes, e.g., to avoid congestion [12], balance the load [4], alleviate interference [13].

In [12], Balachandran et al. focused on the user congestion problem in hot-spot areas of WLANs. They proposed two approaches, namely explicit channel switching and network-directed roaming, to alleviate the congestion in WLANs. Load balancing is considered in [4], from different metrics including the time fairness and min-max fairness. In [4], Bejerano et al. proved that for the time fairness user association problem, there exists a polynomial-time optimal algorithm, while for the min-max fairness bandwidth allocation problem, they designed an approximation algorithm with constant approximation ratio. User association towards proportional fairness (PF) is studied in [7,8]. In some studies [7,8], the PF fairness user association problem was formulated as an integer nonlinear programming problem and is proved to be NP-hard. Due to the hardness of the origin PF fairness association problem, the nonlinear objective function is discretized and relaxed, and then solved by two approximation algorithms. Besides user-to-AP association, [13,14] explored frequency selection for mobile users to minimize interference during user association.

Different methods and techniques are investigated to optimally associate users to APs. The user association problem is modeled and treated as an evolutionary game problem in [15]. Karimi et al. utilized a collaborative method for user association in [16]. Amer et al. applied the realistic media sharing method to improve the network throughput using the 802.11 Distributed Coordination Function (DCF) mode in [17]. They proposed a local search algorithm within neighborhoods to solve the user association problem.

Association control is also explored in other types of networks, such Wireless Mesh Networks (WMN) [18], Multiple Input Multiple Output (MIMO) networks [19] and cellular networks [20]. Furthermore, some researchers considered the dynamics and mobility in WLANs. In [21], Wong et al. suggested re-associating users among neighboring APs when the AP capacity constraints are violated due to the evolving network and users’ movements.

Despite the existing research work on user association and bandwidth allocation in WLANs, very few work consider users’ bandwidth demands. Tang et al. [22] took the users’ bandwidth demands as a new constraint when associating users to APs. However, they assumed that the maximum allocated bandwidth of a user cannot exceed the bandwidth required by this user. In other words, the users’ requirements would be partially satisfied in the proposed scenario. In this paper, we argue that the users’ bandwidth demands must be fully satisfied to ensure the quality of service and mobile users’ experiences. In addition, different from all the above-mentioned work, we consider the perspective of E-WLAN’s providers in saving the network operation cost. Therefore, we target to minimize the E-WLAN operation cost, specifically bandwidth cost by properly associating users to APs while satisfying users’ individual bandwidth demands.

## 3. Problem Formulation and Graph Modeling

In this section, we describe the system model and give some basic assumptions, followed by formulating the bandwidth cost minimization via user association. The notations used in this section is listed in Table 1.

We follow the assumptions and modeling in [3,22]. Consider an E-WLAN consisted of a set of *n* APs denoted by U={1,⋯,n} and a central controller which collects state information from all APs. The union area of the communication ranges for all APs forms the coverage area of the E-WLAN. A set *V* of user devices, V={1,⋯,m}, are within the coverage of the E-WLAN. Assume each user *j* in *V* can be covered by at least one AP and all APs operate at orthogonal channels.

Each user device communicates with APs in its communication range by sending request packets to AP. These requests can be re-routed to the controller in the E-WLAN. As in [22], we can use the size of downlink data arrived in the controller to represent the user demand.

Let $\gamma_{ij}$ denote the experienced SINR of device *j* associated with AP *i*. Then $\gamma_{ij}$ is defined as follows [23].

(1) $$\gamma_{ij}=\frac{t_{i}d_{ij}^{-\alpha }}{N_{0}}$$

$t_{i}$ is the fixed transmission power of AP *i*. $d_{ij}$ is the physical distance between device *j* and AP *i*. α is the shadowing factor ranging from 2.0 to 5.0. $N_{0}$ is the noise in the environment. Then, the effective transmission rate of device *j* that is associated with AP *i*, $b_{ij}=Blog_{2}\left(1+\gamma_{ij}\right)$, where *B* is the channel bandwidth.

**Figure 1:**
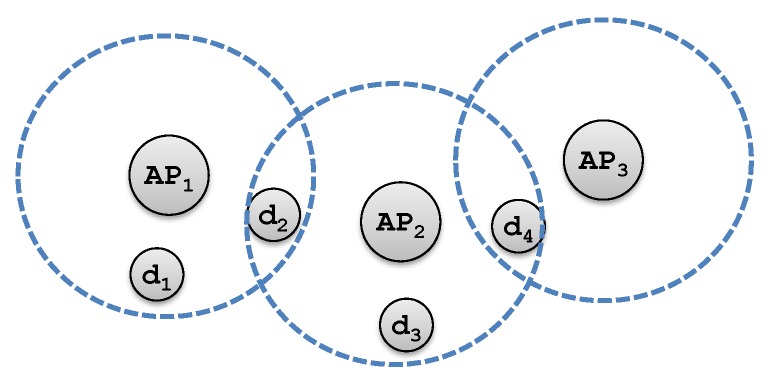
An instance of APs and user devices.

When an AP *i* is in the communication range of a device *j*, AP *i* becomes a candidate AP for device *j*. For example, in Figure 1, AP 1 and AP 2 are candidate APs for device 2. Although each user device may have multiple candidate APs for an association, it can only be associated by at most one AP. We refer this constraint as the *association constraint*. Assume the channels of various AP are orthogonal. Then each user will not be interfered by other APs, and the noise only includes Gaussian white noise [22].

In addition, each AP *i* is associated with a bandwidth capacity $C_{i}$. Each user device *j* can specify a fixed amount of minimal bandwidth $b_{j}$ to support its applications. Also we assume $b_{j}\geq 1$ which indicates each user demands at least 1 unit bandwidth. Next, we present the preliminary definitions and introduce the *Bandwidth Cost Minimization for Association* (BCMA) problem.

**Definition 1** (Assignment P)**.** *An assignment P is a sequence of pairs between APs U and devices V, i.e., P={(i,j)|1≤i≤m,1≤j≤n} such that the association constraint is satisfied.*

If device *j* is associated to AP *i*, then AP *i* must allocate at least the bandwidth of $b_{j}$ to device *j* such that device *j*’s bandwidth demand is satisfied.

**Definition 2** (Bandwidth of Assignment P)**.** *Define the bandwidth of assignment P, $B_{P}$, by the total allocated bandwidth of all assigned devices in P, i.e., $B_{P}=\sum_{j}b_{j}$, j∈{j|(i,j)∈P}.*

Given a set of devices *V* and a set of APs *U* in an enterprise WLAN, the *Bandwidth Cost Minimization for Association (BCMA)* problem is to seek an assignment P so that the bandwidth cost of assignment P is minimized while satisfying the user’s bandwidth demands.

For the bandwidth cost, we consider a simple yet realistic unit cost model [24]. The operator of an enterprise WLAN is usually charged with unit cost model by the ISP (Internet Service Provider). That is, the bandwidth cost is the unit cost (dollars per Mbps) multiplied the total allocated bandwidth. Let *w* be the unit cost for the bandwidth.

If device *j* is associated with AP *i*, let binary variable $x_{ij}=1$; otherwise $x_{ij}=0$. The minimal bandwidth demand $b_{j}$ of user *j* is fixed. Define the effective transmission time between device *j* and AP *i* to be $p_{ij}$. Then effective bit rate of user device *j* to AP *i* is $b_{ij}$. Here, the time demand $p_{ij}$ of user *j* is a variable. We further assume that the unit cost for the bandwidth is *w*. Then, the BCMA problem can be formulated as the following mixed integer optimization problem:

$$\begin{array}{cc} \min\; & \sum_{i\in U}\sum_{j\in V}x_{ij}b_{ij}p_{ij}w \end{array}$$

(2) $$\begin{array}{cccc} s.t.\; & \sum_{j\in V}x_{ij}b_{ij}p_{ij}\leq C_{i}, & \; & \forall i\in U \end{array}$$

(3) $$\begin{array}{cc} \sum_{i\in U}x_{ij}\leq 1, & \forall j\in V \end{array}$$

(4) $$\begin{array}{ccc} \; & \sum_{i\in U}x_{ij}b_{ij}p_{ij}\geq b_{j}, & \forall j\in V \end{array}$$

(5) $$\begin{array}{ccc} \; & x_{ij}\in \left\{0,1\right\}, & \forall i\in U,j\in V \end{array}$$

(6) $$\begin{array}{ccc} \; & p_{ij}\in \left[0,1\right], & \forall i\in U,j\in V \end{array}$$

The constraint in Equation (2) ensures that the total effective rate is capped by each AP’s capacity. The constraint in Equation (3) guarantees that each device can be associated with at most one AP. The constraint in Equation (4) guarantees the minimum transmission rate demand $B_{j}$ for each user *i* is satisfied. The constraint in Equation (6) specifies the time allocated to device *j*. Note that we model the BCMA problem in a unit time.

## 4. Approximation Algorithm Design

In this section, we propose an approximation algorithm via relaxation and rounding method [25] to solve the BCMA problem. The basic idea is to relax the binary variable $x_{ij}$ for allowing the fractional association between devices and APs. Then we obtain an integral association using the rounding technique. Finally, we prove that this method yields a constant approximation ration of 2.

### 4.1. BCMA Problem Relaxation

Note that the relaxed optimization problem may induce a large integrality gap [26,27], i.e., the ratio between the relaxed problem’s result and original problem’s result could be vast or even unbounded. This large integrality gap will lead to difficulty for designing an approximation algorithm. To reduce the integrality gap induced by relaxation, we introduce an additional function h(X,P) to the original objective function. The function h(X,P) is defined as follows:

(7) $$h\left(X,P\right)=\sum_{i\in U}\sum_{j\in V}x_{ij}\sqrt{b_{ij}}p_{ij}^{2}$$

Let $f\left(X,P\right)=\sum_{i\in U}\sum_{j\in V}x_{ij}b_{ij}p_{ij}w$ be the original objective function. Define the new objective function by Γ(X,P)=f(X,P)+h(X,P), where *X* is the matrix of the binary variables $x_{ij}$ and *P* is the matrix of variable $p_{ij}$ for the allocated transmitted time in a unit time. By relaxing the binary variable $x_{ij}$ in Γ(X,P) to the variable with range [0,1], we have the following new relaxed problem which we refer to as the *R-BCMA* problem:

$$\begin{array}{cc} \min\; & \sum_{i\in U}\sum_{j\in V}x_{ij}b_{ij}p_{ij}+\sum_{i\in U}\sum_{j\in V}x_{ij}\sqrt{b_{ij}}p_{ij}^{2} \end{array}$$

(8) $$\begin{array}{cccc} s.t.\; & \sum_{j\in V}x_{ij}b_{ij}p_{ij}\leq C_{i}, & \; & \forall i\in U \end{array}$$

(9) $$\begin{array}{ccc} \; & \sum_{i\in U}x_{ij}\leq 1, & \forall j\in V \end{array}$$

(10) $$\begin{array}{ccc} \; & \sum_{i\in U}x_{ij}b_{ij}p_{ij}\geq b_{j}, & \forall j\in V \end{array}$$

(11) $$\begin{array}{ccc} \; & x_{ij}\in \left[0,1\right], & \forall i\in U,j\in V \end{array}$$

(12) $$\begin{array}{ccc} \; & p_{ij}\in \left[0,1\right], & \forall i\in U,j\in V \end{array}$$

In the R-BCMA problem, the objective function and the constraints in Equations (8) and (10) are convex and other constraints are linear. The R-BCMA problem is a typical convex optimization problem which could be solved by with the desired precision in polynomial time by corresponding algorithms [28,29], for example, interior-point methods [28].

With the new additional objective function h(X,P) in the R-BCMA problem, we show that the integrality gap induced by the relaxation is reduced to a constant bound of 2.

**Lemma** **1.** *Let $\left(X^{★},P^{★}\right)$ and $\left(\tilde{X},\tilde{P}\right)$ be the optimal solutions to the original BCMA problem and its relaxed R-BCMA problem, respectively. Then, $\Gamma \left(\tilde{X},\tilde{P}\right)\leq 2f\left(X^{★},P^{★}\right)$.*

**Proof.**  Since $\left(X^{★},P^{★}\right)$ is the optimal solution to the original BCMA problem, $\left(X^{★},P^{★}\right)$ is also a feasible solution to the its relaxed R-BCMA problem. Note that the new added function h(X,P) does not affect the feasibility of $\left(X^{★},P^{★}\right)$ to the R-BCA problem. Therefore, we have $\Gamma \left(\tilde{X},\tilde{P}\right)\leq f\left(X^{★},P^{★}\right)+h\left(X^{★},P^{★}\right)$, since R-BCA problem is a minimization optimization problem. According the definitions of functions f(X,P) and h(X,P), we have $h\left(X^{★},P^{★}\right)\leq f\left(X^{★},P^{★}\right)$, since $p_{ij}\in \left[0,1\right]$ and $b_{ij}\geq 1$. Thus, $\Gamma \left(\tilde{X},\tilde{P}\right)\leq f\left(X^{★},P^{★}\right)+h\left(X^{★},P^{★}\right)\leq f\left(X^{★},P^{★}\right)+f\left(X^{★},P^{★}\right)=2f\left(X^{★},P^{★}\right)$. □

### 4.2. Obtaining Integral Solution by Rounding

Although the added objective function h(X,P) reduces the integrality gap, the association result *X* indicating the pairs between user devices and APs is fractional, i.e., $\left\{0\leq x_{ij}\leq 1|x_{ij}\in X\right\}$. In this subsection, we adopt the rounding technique [25] to achieve the integral association. The idea is to create a bipartite graph based on the resulting *X* with a fractional association and then find a minimum total bandwidth matching. The resulting matching corresponds to an integral solution. Details are illustrated as follows.

First, we construct a bipartite graph *G*, which is defined in Definition 3.

**Definition 3** (Bipartite Graph *G* for Rounding)**.** *Given the fractional solution $\tilde{X}$ and $\tilde{P}$ of R-BCMA problem, a bipartite graph G=(L,R,E,W,C) for rounding is constructed by the following steps:*

*(1)*  *For each device j∈V, create a corresponding vertex $v_{j}\in R$ in G.*
*(2)*  *For each AP i∈U, let $k_{i}=\left\lceil \sum_{j}x_{ij}\right\rceil$. Create $k_{i}$ vertices in L, i.e., $L=\{u_{iq}|i\in \left[1,m\right],q\in \left[1,k_{i}\right]\}$.*
*(3)*  *If $\sum_{j}x_{ij}\leq 1$, there is only one vertex $u_{i1}$ for AP node i. Create edge $\left(u_{i1},v_{j}\right)$ in E and set its weight $w\left(u_{i1},v_{j}\right)=x_{ij}$ and cost $c\left(u_{i1},v_{j}\right)=b_{ij}p_{ij}$.*
*(4)*  *If $\sum_{j}x_{ij}\geq 1$, $\forall q\in [1,k_{i}-1]$, let $\left\{j_{q}|q=1,2,\cdots ,k_{i}-1\right\}$ be the minimum index such that $\sum_{j=1}^{j_{q}}x_{ij}\geq q$. For convenience, we define $j_{0}=0$. Create edges $\left\{\left(u_{iq},v_{j}\right)|q\in \left[1,k_{i}-1\right],j\in \left[j_{q-1}+1,j_{q}-1\right]\right\}$ in E. Assign its weight $w\left(u_{iq},v_{j}\right)=x_{ij}$ and cost $c\left(u_{iq},v_{j}\right)=b_{ij}p_{ij}$.*
*(5)*  *Create edge $\left(u_{iq},v_{j_{q}}\right)$ in E and assign its weight $w\left(u_{iq},v_{j_{q}}\right)=1-\sum_{j=j_{q-1}+1}^{j_{q}-1}w\left(u_{iq},v_{j}\right)$. Create edge $(u_{i(q+1)},v_{j_{q}})\in E$ and assign its weight $w\left(u_{i(q+1)},v_{j_{q}}\right)=x_{ij_{q}}-w\left(u_{iq},v_{j_{q}}\right)=\sum_{j=1}^{j_{q}}x_{ij}-q$ and cost $c\left(u_{i(q+1)},v_{j_{q}}\right)=b_{ij_{q}}p_{ij_{q}}$.*
*(6)*  *For $q=k_{i}$ and $j\in (j_{k_{i}-1},j_{k_{i}}]$, create edge $\left(u_{iq},v_{j}\right)$ and assign its weight $w\left(u_{iq},v_{j}\right)=x_{ij}$ and cost $c\left(u_{iq},v_{j}\right)=b_{ij}p_{ij}$.*

Note that the weight and the cost for each edge in the construction of *G* are also assigned. From the above steps, each AP *i* actually corresponds to $k_{i}$ nodes in the bipartite graph *G*. For each corresponding node $u_{iq}$, some properties of *G* are described in as follows.

**Property** **1.** *For each of AP i’s corresponding node $u_{iq}\in G$ ($q=1,2,\cdots ,k_{i}-1$), the total weight of all its incident edges is equal to 1, i.e., $\sum_{j=j_{q-1}+1}^{j_{q}}w\left(u_{iq},v_{j}\right)=1$.*

**Proof.**  Since $\left\{j_{q}|q=1,2,\cdots ,k_{i}-1\right\}$ is the minimum index such that $\sum_{j=1}^{j_{q}}x_{ij}\geq q$, we have $\sum_{j=j_{q-1}+1}^{j_{q}-1}x_{ij}\leq 1$. In step 4, for each $u_{iq}$ and $j\in [j_{q-1}+1,j_{q}-1]$, the $x_{ij}$ is assigned to $\left(u_{iq},v_{j}\right)$. In step 5, the value $w\left(u_{iq},v_{j_{q}}\right)=1-\sum_{j=j_{q-1}+1}^{j_{q}-1}w\left(u_{iq},v_{j}\right)$ is assigned. This property follows. □

**Property** **2.** *For each of AP i’s corresponding node $u_{ik_{i}}$, the total weight of all its incident edges $\sum_{j=j_{k_{i}}-1}^{j_{k_{i}}}w\left(u_{ik_{i}},v_{j}\right)\leq 1$.*

**Proof.**  In step 6 of the construction of *G*, it constructs the edges for the last corresponding node $u_{ik_{i}}$ for each AP *i*. Since $k_{i}=\left\lceil \sum_{j}x_{ij}\right\rceil$ and $\left\{j_{q}|q=1,2,\cdots ,k_{i}-1\right\}$ is the minimum index such that $\sum_{j}^{j_{q}}x_{ij}\geq q$, we have $\sum_{j=j_{k_{i}-1}}^{j_{k_{i}}}x_{ij}\leq 1$. According to step 5, $w\left(u_{ik_{i}},j_{k-1}\right)<x_{ij_{k-1}}$. According to step 6, the weight of incident edges $\left\{w\left(u_{ik_{i}},v_{j}\right)=x_{ij}|j\in \left(j_{k_{i}-1},j_{k_{i}}\right]\right\}$. Thus this property is proved.

Step 3 considers the case that $\sum_{i}x_{ij}\leq 1$, then their is only one corresponding node $u_{i1}$ for AP *i*. According to the weight assignment in this step, this property still follows. □

Second, we find the minimum cost matching M on the constructed bipartite graph *G*. The resulting matching *M* corresponds to an AP-device association solution. That is, for each edge $(u_{iq},v_{j})\in M$, let device *j* associate AP *i*.

### 4.3. Algorithm Analysis

The algorithm for the BCMA problem is summarized in Algorithm 1, which is referred to as the *RAA* algorithm.

**Algorithm 1** Rounding Algorithm for Association (RAA)
1:Solve the R-BCMA problem to obtain the resulting matrices $\tilde{X}=\left\{x_{ij}|i\in U,j\in V\right\}$ and $\tilde{P}=\left\{p_{ij}|i\in U,j\in V\right\}$2:Construct the graph *G* with $\tilde{X}$ and $\tilde{P}$ by Definition 3.3:Apply minimum cost flow algorithm [30] to find a maximum matching M with minimum cost on graph *G*. 4:Each edge in $(u_{iq},v_{j})\in M$ corresponds to an association between AP *i* and device *j*, i.e., $\hat{x}\left(i,j\right)=1$, otherwise $\hat{x}\left(i,j\right)=0$. Let matrix $\hat{X}=\left\{\hat{x}\left(i,j\right)=1|i\in U,j\in V\right\}$ denote the final integral association result. 5:Return $\hat{X}$.


Let $f(\hat{X},\tilde{P})$ be the final result for BCMA problem. Next, we prove the approximation ratio of the RAA algorithm.

**Lemma** **2.** *Let AP r be the AP which is associated with device j in the final solution $\hat{X}$, i.e., $\hat{x}\left(r,j\right)\in \hat{X}$. Let $\left(u_{rq},v_{j}\right)$ be the edge corresponding to $\hat{x}\left(i,j\right)=1$ in M. Then, ∀j∈V, $c(u_{rq},v_{j})$ is the minimum cost among all costs between device j and any other AP i, i∈U∩{i≠r}.*

**Proof.**  For the device *j*, the case of $x_{ij}=1$ for an AP node *i*: From steps 4 and 5 in the construction of *G*, there may be more than one edge incident to device *j* from nodes $u_{iq}$, i.e., $\sum_{q}x\left(u_{iq},j\right)=1$. However, for the device *j* and AP node *i*, the cost $c(u_{iq},v_{j})$ of edges incident to device *j* from nodes $u_{iq}$, $q\in [1,k_{i}]$, are all equal to $b_{ij}p_{ij}$. Since one of the edges incident to node $v_{j}$ must be selected into the resulting matching M. Thus the lemma holds.

For the device *j*, the case of $\sum_{i}x_{ij}=1$ and $x_{ij}<1$: Since the matching with minimum cost is selected on graph *G*, then for each device *j*, only one edge incident to node $v_{j}$ is selected in the final matching M. Assume this selected edge is $\left(u_{rq},v_{j}\right)$ on *G*. We argue that this edge $\left(u_{rq},v_{j}\right)$ has the minimum cost among all costs between between device *j* and any other AP *i*, i∈U∩i≠r. Assume this edge $\left(u_{rq},v_{j}\right)$ does not have the minimum cost. Then M must not be the matching with the minimum cost, since we can replace the edge $\left(u_{rq},v_{j}\right)$ with the edge with minimum cost between device *j* and any other AP *i*, i∈U∩i≠r and obtain a matching $M^{\prime }$ with less cost. This contradicts with the fact that M is the matching with the minimum cost. Thus the lemma holds. □

**Lemma** **3.** *$\sum_{i}\sum_{j}{\hat{x}}_{ij}b_{ij}p_{ij}\leq \sum_{i}\sum_{j}{\tilde{x}}_{ij}b_{ij}p_{ij}$*

**Proof.**  For the device *j*, consider the first case that ${\tilde{x}}_{rj}=1$ for an AP node *r*: From steps 4 and 5 in the construction of *G*, we can see at most two edges incident to device *j* from nodes $u_{rq}\in L$ on graph *G*. Note that according to the construction steps, we have $\exists r\exists q,\sum_{r}\sum_{q}w\left(u_{rq},v_{j}\right)={\tilde{x}}_{rj}=1$ for the device *j*. Let the $E_{j}^{1}=\left\{\left(u_{rq},v_{j}\right)|\sum_{r}\sum_{q}w\left(u_{rq},v_{j}\right)=1,\exists r\exists q,\right\}$ be the set of at most two such edges. For the device *j* and its incident AP nodes $u_{rq}$, the cost $c(u_{rq},v_{j})$ of the edges in $E_{j}^{1}$ are the same which is equal to $b_{rj}p_{rj}$. Thus, for the device *j*, since $\sum_{r}\sum_{q}\tilde{x}\left(u_{rq},v_{j}\right)=1$, we have

(13) $$\sum_{r}\sum_{q}\tilde{x}\left(u_{rq},v_{j}\right)*b_{rj}p_{rj}=b_{rj}p_{rj}$$

Note that one of the edges in $E_{j}^{1}$ must be selected into the resulting matching M, otherwise M is not a maximum matching with minimum cost. For the device *j*, then we have $\sum_{i}\hat{x}\left(i,j\right)b_{ij}p_{ij}=b_{rj}p_{rj}$, since $\hat{x}\left(i,j\right)=1$. Then, for the case that $\tilde{x}\left(r,j\right)=1$, we have the lemma proved.

For the device *j*, consider the second case that $\sum_{i}{\tilde{x}}_{ij}=1$, i.e., there are more than one AP nodes which are fractionally associated with the device *j*. Let the $E_{j}^{2}=\left\{\left(u_{iq},v_{j}\right)|\sum_{i}\sum_{q}w\left(u_{iq},v_{j}\right)=1\right\}$ be the set of the edges incident to the device node $v_{j}$ in *G*.

Also note that there must be exactly one edge in $E_{j}^{2}$ which is selected into the the resulting matching M. Let this selected edge be $\left(u_{rq},v_{j}\right)$. Then for the device *j*, its final cost is

(14) $$\begin{array}{c} f_{j}\left(\hat{X},\tilde{P}\right)={\hat{x}}_{rj}c\left(u_{rq},v_{j}\right)=c\left(u_{rq},v_{j}\right) \end{array}$$

The fractional cost for the device *j* before rounding procedure is

(15) $$\begin{array}{c} f_{j}\left(\tilde{X},\tilde{P}\right)=\sum_{q}w\left(u_{rq},v_{j}\right)c\left(u_{rq},v_{j}\right)+\sum_{i\neq r,}^{i=|U|}\sum_{q^{\prime }}w\left(u_{iq^{\prime }},v_{j}\right)c\left(u_{iq^{\prime }},v_{j}\right),\exists r,q\in \left[1,k_{r}\right],q^{\prime }\in \left[1,k_{i}\right] \end{array}$$

Since

(16) $$\sum_{q}w\left(u_{rq},v_{j}\right)+\sum_{i\neq r,}^{i=|U|}\sum_{q^{\prime }}w\left(u_{iq^{\prime }},v_{j}\right)=1,$$

we have the following:

(17) $$\begin{array}{cc} f_{j}\left(\tilde{X},\tilde{P}\right)-f_{j}\left(\hat{X},\tilde{P}\right) & =\left(\sum_{q}w\left(u_{rq},v_{j}\right)-1\right)c\left(u_{rq},v_{j}\right)+\sum_{i\neq r,}^{i=|U|}\sum_{q^{\prime }}w\left(u_{iq^{\prime }},v_{j}\right)c\left(u_{iq^{\prime }},v_{j}\right)\\ & =\left(-\sum_{i\neq r,}^{i=|U|}\sum_{q^{\prime }}w\left(u_{iq^{\prime }},v_{j}\right)\right)c\left(u_{rq},v_{j}\right)+\sum_{i\neq r,}^{i=|U|}\sum_{q^{\prime }}w\left(u_{iq^{\prime }},v_{j}\right)c\left(u_{iq^{\prime }},v_{j}\right) \end{array}$$

According to Lemma 2, we have

(18) $$\begin{array}{c} c\left(u_{rq},v_{j}\right)\leq c\left(u_{iq^{\prime }},v_{j}\right),\forall i\in U\cap \left\{i\neq r\right\} \end{array}$$

Then for each *j*, we have

(19) $$\begin{array}{c} f_{j}\left(\tilde{X},\tilde{P}\right)-f_{j}\left(\hat{X},\tilde{P}\right)\geq 0 \end{array}$$

Then,

(20) $$\begin{array}{c} \sum_{j}f_{j}\left(\tilde{X},\tilde{P}\right)\geq \sum_{j}f_{j}\left(\hat{X},\tilde{P}\right)\Rightarrow \sum_{i}\sum_{j}{\tilde{x}}_{ij}b_{ij}p_{ij}\geq \sum_{i}\sum_{j}{\hat{x}}_{ij}b_{ij}p_{ij} \end{array}$$

That is $f\left(\tilde{X},\tilde{P}\right)\geq f\left(\hat{X},\tilde{P}\right)$. □

**Theorem** **1.** *The approximation ratio of the proposed RAA algorithm is 2.*

**Proof.**  According to lemma 1, we have $\Gamma \left(\tilde{X},\tilde{P}\right)\leq 2f\left(X^{★},P^{★}\right)$. By lemma 3, we have $f\left(\hat{X},\tilde{P}\right)\leq \Gamma \left(\tilde{X},\tilde{P}\right)\leq 2f\left(X^{★},P^{★}\right)$. Since $f(\hat{X},\tilde{P})$ is the solution of our proposed RAA algorithm and $f(X^{★},P^{★})$ is the optimal solution of the BCMA problem, the theorem is proved. □

**Theorem** **2.** *The time complexity of the proposed RAA algorithm is $O(m^{2}n^{2}log\left(n\right))$.*

**Proof.**  The time complexity for our RAA algorithm can be analyzed as follows. Its overall complexity is composed by three parts, which are the complexity of solving the relaxed BCMA problem, the complexity of constructing bipartite graph and computing the minimum cost value over the constructed bipartite graph. The complexity for solving the relaxed BCMA optimization problem is determined by the problem size which is $O(m^{2}n^{2})$. Thus, the complexity for this part is $\sqrt{m^{2}n^{2}}ln\left(1/\epsilon \right)$ [29]. The bipartite graph construction needs $O(mn^{2})$ time complexity, since each corresponding AP node may construct up to *n* auxiliary nodes. The applied min-cost algorithm for the minimum cost value computation needs time complexity $O(m^{2}n^{2}log\left(n\right))$ using minimum-mean cost cycle canceling algorithm [30]. Thus, the overall time complexity is dominated by the min-cost algorithm which is $O(m^{2}n^{2}log\left(n\right))$. □

## 5. Performance Evaluation

We investigated the performance of our algorithm *RAA* for the BCMA problem. Since there is no similar work on the revenue minimization with considering users’ bandwidth demands, we utilized the network flow algorithm in [3] (referred as *NetFlow* algorithm) as the benchmark to demonstrate the superiority of our algorithm. We compared our RAA algorithm with NetFlow algorithm in terms of minimum cost ( referred as min-cost) which is the value of our objective function. In addition, we show the corresponding throughput comparison results when the min-cost results are achieved. Moreover, due to bandwidth capacity constraint, some mobile users may not be able to be associated with any AP. We utilized user acceptance ratio to denote the percentage of users who have been associated. To further show the superiority of our algorithm, we also compared our RAA algorithm with NetFlow algorithm in the aspect of the user acceptance ratio.

Our simulated network consisted of 10 APs placed on a 20 × 20 grid. The AP’s coverage was set to 10 m. For each generated instance, all user devices were randomly generated within the grid. Similar to [22], we set the transmission power $t_{i}$ of each AP *i* as 20 dBm, the shadowing factor α as 4 and the noise power $N_{0}$ as −80 dBm. The user bandwidth demand $b_{j}$ was randomly generated within the range [10,20] for each user *j*. AP *i*’s bandwidth capacity $C_{i}$ was randomly generated within the range [40,100]. For simplicity, we set the unit cost for the bandwidth *w* as 1.

### 5.1. Performance of the RAA Algorithm for the BCMA Problem

**Figure 2:**
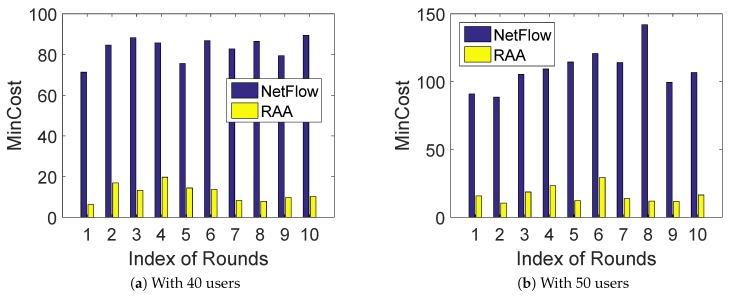
MinCost comparison for 10 rounds.

**Figure 3:**
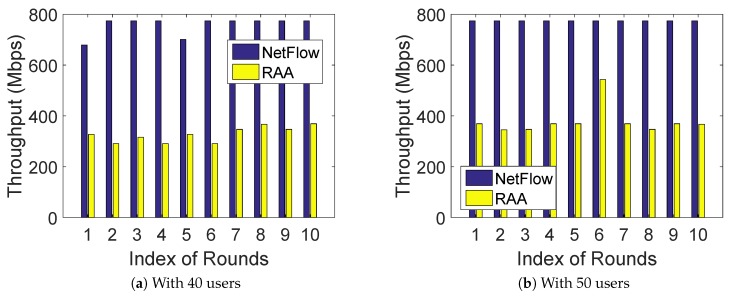
Throughput comparison for 10 rounds.

To compare the performance of the algorithm, we first ran both RAA and NetFlow algorithms 10 times under a random deployment with a fixed number of users. Figure 2a,b presents the min-cost result for both algorithms when the number of *m* users was fixed at 40 and 50, respectively. As shown in Figure 2a,b, our RAA algorithm always performed much better than that of NetFlow algorithm. Moreover, our algorithm only generated about 1/7 min-cost of the NetFlow algorithm on average for the total 10 rounds. Figure 3a,b presents the corresponding throughput result for both algorithms when the number of users was fixed with 40 and 50 respectively. The throughput comparison result shown in Figure 3a,b is consistent with the min-cost result, since less throughput leads to less cost.

**Figure 4:**
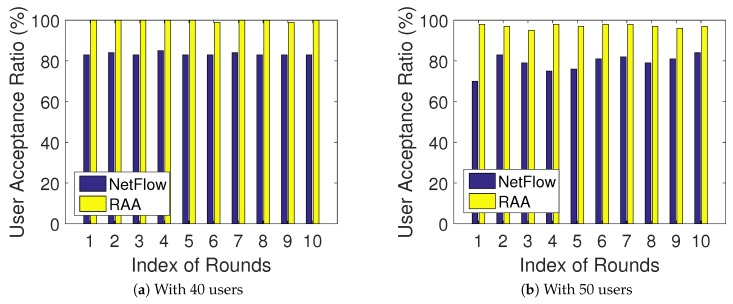
User acceptance ratio comparison for 10 rounds.

Figure 4a,b presents the user acceptance ratio results for both algorithms when the number of *m* users was fixed with 40 and 50, respectively. When the number of users was 40, we can see in Figure 4a that our RAA algorithm achieved 100% user acceptance ratio in eight rounds and 99% in the remaining two rounds. When the number of users was 50, Figure 4b shows that our algorithm achieved almost 100% user acceptance ratio in every round, while the NetFlow algorithm could only achieve around 80% user acceptance ratio. Overall, our algorithm performed much better in the aspect of user acceptance ratio.

### 5.2. Impact of the Number of Users

**Figure 5:**
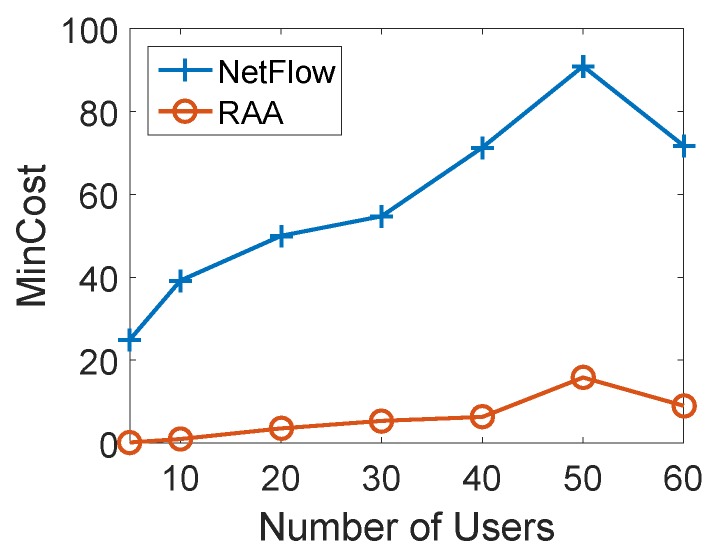
Mincost for varied numbers of users with 10 APs.

We studied the impact of the number of users for both algorithms. We first fixed the number of APs as 10 and gradually increased the number of user devices from 5 to 60 to compare the total min-cost and user acceptance ratio between two algorithms. For each load, we ran both algorithms 10 times with the fixed number of mobile users to get the average results. Figure 5 depicts the result of the average total min-cost for both algorithms as the number of users varies from 10 to 60 with an increment of 10. When the number of users was 40, the average min-cost of the NetFlow algorithm was 71.35, while it was only 6.29 for our RAA algorithm. From the curves in Figure 5, we can see that the cost of our algorithm gradually increased while the cost of the NetFlow algorithm grew sharply when the number of users increased.

**Figure 6:**
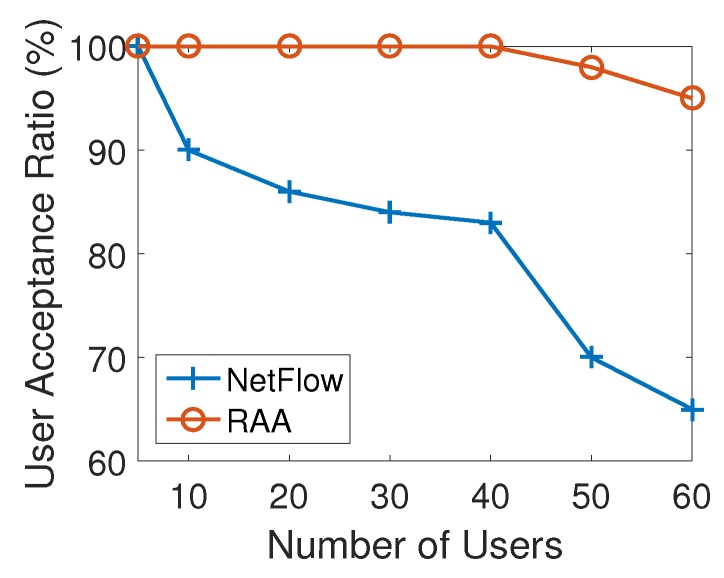
User acceptance ratio for varied numbers of users with 10 APs.

**Figure 7:**
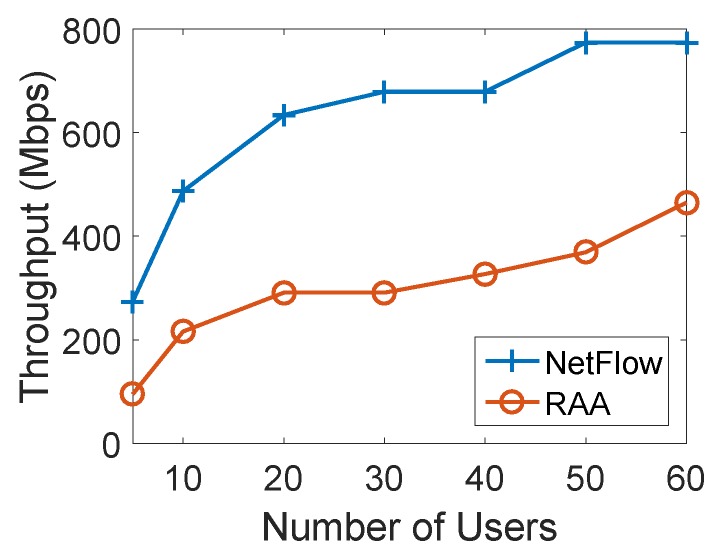
Throughput for varied numbers of users with 10 APs.

Figure 6 presents how the average user acceptance ratio produced by RAA and NetFlow algorithms change when the number of users increased from 5 to 60 with an increment of 10. As the number of users increased, the user acceptance ratio from both algorithms decreased. This result is consistent with the expectation that there will be some users that will not be able to be associated since the capacities of APs are limited. However, from the result of Figure 6, our RAA algorithm accepted many more users as the number of users increases. In addition, our algorithm’s user acceptance ratio dropped very slowly as the number of users increases, while the NetFlow algorithm’s result droped sharply. When the number of users reached 60, the user acceptance ratio from NetFlow algorithm has dropped to 65% while the RAA algorithm’s ratio could still be 95%. Figure 7 presents the throughput generated by both algorithms. This result also shows that our algorithm produced much less throughput which induces the less cost as shown in Figure 5.

**Figure 8:**
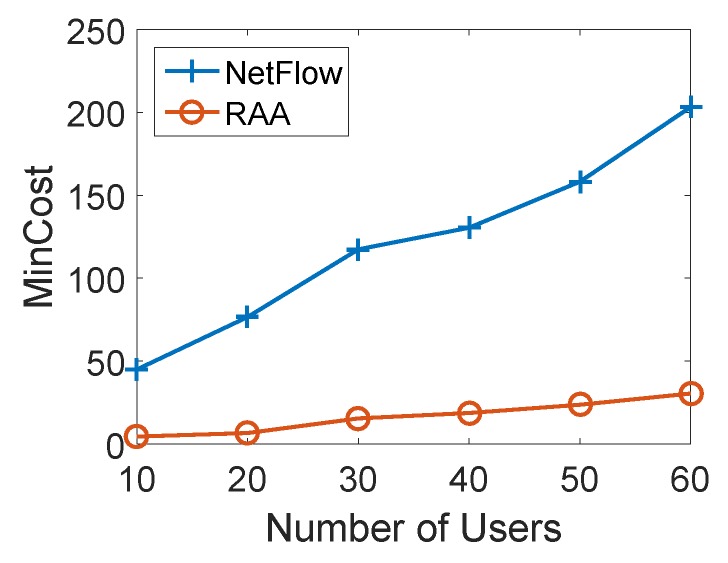
Mincost for varied numbers of users with 20 APs.

**Figure 9:**
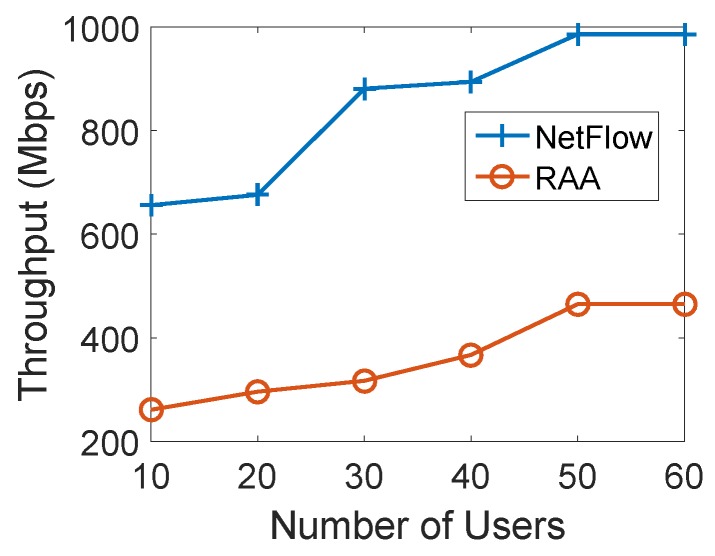
Throughput for varied numbers of users with 20 APs.

**Figure 10:**
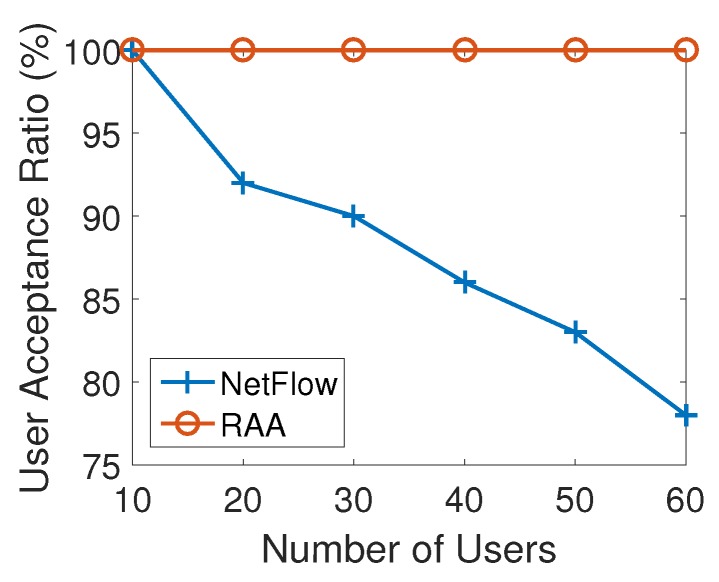
User acceptance ratio for varied numbers of users with 20 APs.

To further validate the above observation, we also checked the performance of both algorithms when the number of APs was 20 with increasing users from 10 to 60. The min-cost, throughput and user acceptance ratio are shown in Figure 8, Figure 9 and Figure 10, respectively. From these results, we can conclude that the same observations hold.

**Figure 11:**
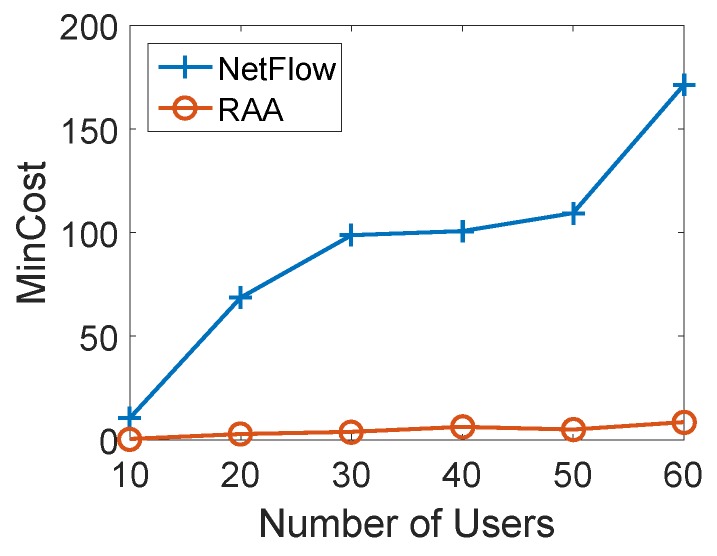
Mincost with non-uniform distribution.

**Figure 12:**
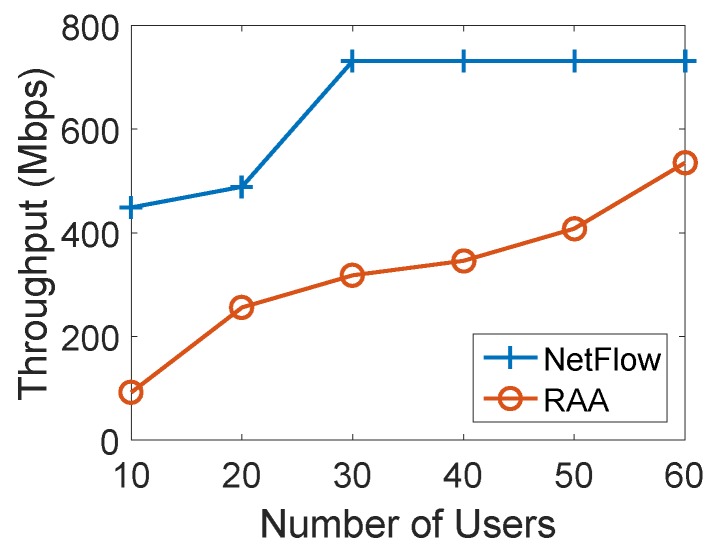
Throughput with non-uniform distribution.

**Figure 13:**
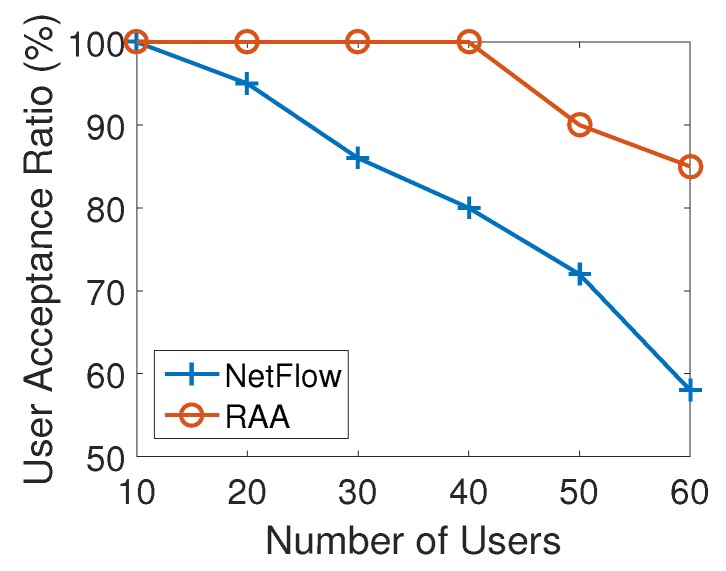
User acceptance ratio with non-uniform distribution.

We also tested the performance of our algorithm when the users were non-uniformly distributed to simulate the hotspot scenario. Figure 11 presents the min-cost results for both algorithms with 10 APs when the users were generated under a non-uniform distribution. From this result we can see that our algorithm maintained the very low cost even with the increasing number of users, while the min-cost of the NetFlow algorithm increased very sharply. This result shows that our algorithm could always achieve a small cost under the non-uniform user distribution. Figure 12 and Figure 13 shows the min-cost and user acceptance ratio results for both algorithms with 10 APs when the users were generated under a non-uniformly distribution, respectively. Figure 12 tells us that our algorithm always achieved much less throughput than that of NetFlow algorithm. When the number of users was 60, our algorithm still achieved an 85% acceptance ratio while the NetFlow algorithm could only achieve 58%. Figure 13 shows that user acceptance ratio of our algorithm could be maintained at a high level even when the number of users increased under the non-uniform distribution.

## 6. Conclusions and Future Work

We have formulated the bandwidth cost minimization problem of scheduling the user association for E-WLANs. To solve this problem, we first introduced an additional objective function to bound the integer gap caused by the integer relaxation. Then we developed an algorithm based on the rounding technique and prove the approximation ratio of the proposed algorithm. Our algorithm achieves the constant approximation ratio for the proposed bandwidth cost minimization problem. Extensive simulations were carried out to further validate the superiority of our proposed algorithm.

We focused on the association with user bandwidth demands under the static settings in this study. As a future research direction, dynamic user demands and behaviors would be taken into consideration. Specifically, we will extend the current model and algorithms to be adaptive to dynamic scenarios. In addition, we will explore non-stable network conditions, e.g, connectivity states and available bandwidth. We plan to apply nonlinear transformation techniques, e.g., discretization and regularization, or convex sum-of-squares relaxations followed by semi-definite programming method to address the non-convex constraints induced by non-stable network factors.

## Figures and Tables

**Table 1:** Notations Used.

Notation	Explanation
*U*	the set of APs in an E-WLAN
*V*	the set of user devices
*n*	the number of APs in set *U*
*m*	the number of user devices in set *V*
*i*	the index of an AP, i∈U
*j*	the index of a user device j∈V
$\gamma_{ij}$	the experienced SINR of device *j* associated with AP *i*
$t_{i}$	the fixed transmission power of AP *i*
$d_{ij}$	the physical distance between device *j* and AP *i*
α	the shadowing factor ranging from 2.0 to 5.0
$N_{0}$	the noise in the environment
$b_{ij}$	the effective transmission rate of device *j* which is associated with AP *i*
*B*	the channel bandwidth
$C_{i}$	the bandwidth capacity of AP *i*
$b_{j}$	the specified minimal bandwidth demand of user device *j* to support its applications
$x_{ij}$	binary variable indicating that if device *j* is associated with AP *i* or not
$p_{ij}$	the effective transmission time between device *j* and AP *i*
*w*	the unit cost for the bandwidth
